# Socio-cultural factors surrounding mental distress during the perinatal period in Zambia: a qualitative investigation

**DOI:** 10.1186/1752-4458-6-12

**Published:** 2012-09-06

**Authors:** Lonia Mwape, Teena M McGuinness, Rachael Dixey, Sally E Johnson

**Affiliations:** 1 Department of Nursing Sciences, University of Zambia, School of Medicine, Box 50110, Nationalist Road, Lusaka, Zambia; 2 Faculty of Nursing, University of Alabama at Birmingham, NB 420, 1720 2nd Avenue South, Birmingham, USA; 3 Faculty of Health and Social Sciences, Leeds Metropolitan University, Calverley Street, Leeds, West Yorkshire, LS 1 3HE, UK; 4 Department of Nursing Sciences, University of Zambia, School of Medicine, Box 50110, Nationalist Road, Lusaka, Zambia

**Keywords:** Perinatal period, Mental distress, Parenting, Psychological distress

## Abstract

**Background:**

The presence of mental distress during pregnancy and after childbirth imposes detrimental developmental and health consequences for families in all nations. In Zambia, the Ministry of Health (MoH) has proposed a more comprehensive approach towards mental health care, recognizing the importance of the mental health of women during the perinatal period.

**Aim:**

The study explores factors contributing to mental distress during the perinatal period of motherhood in Zambia.

**Methods:**

A qualitative study was conducted in Lusaka, Zambia with nineteen focus groups comprising 149 women and men from primary health facilities and schools respectively.

**Findings:**

There are high levels of mental distress in four domains: worry about HIV status and testing; uncertainty about survival from childbirth; lack of social support; and vulnerability/oppression.

**Conclusion:**

Identifying mental distress and prompt referral for interventions is critical to improving the mental health of the mother and prevent the effects of mental distress on the baby.

**Recommendation:**

Strategies should be put in place to ensure pregnant women are screened for possible perinatal mental health problems during their visit to antenatal clinic and referral made to qualified mental health professionals. In addition further research is recommended in order to facilitate evidence based mental health policy formulation and implementation in Zambia.

## Background

The perinatal period has been considered as a time of crisis brought about by emotional, psychological and social stress [1]. For many women this period can be a time of heightened risk for mental health and emotional responses. Women in the perinatal period may be increasingly vulnerable to affective disorders, psychotic illness and psychological distress [2,3]). Exacerbation of pre-existing mental disorders such as depression, that were present in antenatal period or a psychotic illness such as schizophrenia might be experienced. For others, the perinatal period might bring the first experience of mental difficulties and psychological distress [4]. According to Crawford and Unger [5] psychosocial factors such as stressful situations, trait anxiety and life changes have been associated with pregnancy and birth complications such as pre-eclampsia, pre-term labour and prolonged pregnancy.

Additionally, transition to motherhood is said to be associated with a decline in personal well-being and a general increase in distress resulting from the magnitude of adjustments made as a result of the changes that occur during this period: Depressive symptoms, hostility and heightened anxiety have been reported during the transition [6]. Further, [Jomeen and Martin 7], who assessed women in the antenatal period for depression, emphasized the importance of identifying maternal psychological distress during the course of pregnancy as a strategy to prevent postpartum depression. Other studies on antenatal depressive mood have examined antenatal mood as the predictor of postnatal depression [8,9] with psychological disturbance and distress during pregnancy as risk factors.

Postnatal depression has been extensively studied in both the biomedical and social science realms focusing on individual factors, (such as hormones and previous psychiatric morbidity), context and experience of childbirth, and motherhood, respectively [10]. Postnatal depression involves a range of depressive symptoms variable in their severity and duration that may be suffered by a mother after childbirth. The difference between postnatal depression and psychological distress is embedded in the difference in severity of the symptoms exhibited by the patient [11].

In depression, the mother may present with increased levels of anxiety [12], declining levels of satisfaction with spouse, and reduced enjoyment and positive attitude towards the baby [13], over a period of two weeks [11]. In psychological distress similar symptoms may be exhibited but the levels will be lower than those in depression. Both depression and psychological distress can be identified when the cut-off for the measure being used is adjusted to accommodate those with mild symptoms [14]. The global prevalence rate of postnatal depression has been reported in 13 percent of the population, with adverse consequences for women and the family as whole, some cases of which have resulted in maternal deaths through suicide [15]. Suicide accounts for a significant number of deaths in young women in resource constrained countries [16].

Evidence from India and South Africa has shown that psychological distress in the perinatal period impacts on child health and social development [17]. In addition, infants of mothers who are depressed are more likely to be of low birth weight, malnourished, and stunted by the age of six months. Diarrhoeal diseases are also said to be more prevalent in these children. A combination of all these factors may increase the risks of child deaths [3].

The negative impact resulting from distress during the perinatal period on the offspring may include poor mental health outcomes in childhood. Evidence also suggests that early parent-infant interactions directly affect the development of certain key regions of the brain and that repeated psychological trauma in infancy will impair functions in the brain and consequently contribute to the development of post-traumatic stress disorder [18]. Perinatal mental disorders represent substantial social suffering in women [3]. In the context of chronic social adversity, maternal depression is especially detrimental to child development [19].

### Cultural context of psychological distress in the perinatal period

Perinatal mental health is an important index for the reproductive health status of women globally. From a South African view point, perinatal mental health problems have emerged as an epidemic in low-income communities with the prevalence as high as one in every three women [20]. According to Fisher et al. [3], studies conducted in resource-constrained countries have found rates two to three times higher than those found in high-income countries. Turkey, Vietnam, and India presented up to date data demonstrating that 25 percent of mothers experienced psychological distress. Concerns for the health of the mother and her newborn baby exist in all cultural groups in Africa with indigenous practices surrounding perinatal survival and child spacing [21].

Many societies have specific traditional practices applicable to a woman in the few days that follow childbirth. For instance, Jamaican traditions involve an intense nine-day seclusion following childbirth that is followed by a less restrictive thirty one days when the mother remains at home with her baby and is looked after by her own mother.

For many Hindus, a woman is regarded as impure for 40 days after childbirth and during this time, she and her child should not come out of confinement. Many Chinese women, on the other hand, are given extra attention and spared all house chores in the first postpartum month. The woman is attended by a female relative usually her mother or mother-in-law [22].

This confinement, examined from a positive perspective serves as enriched social support albeit a woman is labelled as contaminated in some aspects, and may help curb the development of perinatal depression. Looking at confinement imposed on women after childbirth in the mentioned cultures from a different perspective, it can be contended that the practice may also serve to enhance negative self-concept that may contribute to the development of postnatal psychological distress.

[Affonso and Mayberry 23] suggest that because pregnancy and childbirth have become safer, the emphasis has shifted from factors affecting survival to those concerned with the psychological aspects of the experience. However, this might not be the case in the developing world where the focus might still be around issues of survival. Abiodum [24], writing from Nigeria, asserted that little attention has been paid to the mental health problems experienced by women in the perinatal period in Nigeria and other developing countries. [Guse et al., 25] in their study on the effects of hypnotherapy on postnatal maternal psychological well being in South Africa, argued that psychological research on this period concentrates more on pathological aspects related to pregnancy, childbirth and the postpartum period rather than support around the transition to parenthood.

Similarly, the situation in Zambia regarding perinatal care raises the same concern. Many cases of psychological distress during the perinatal period may go unreported [26].

This is heightened or perpetuated by the absence of a deliberate policy to screen for depression during antenatal and postnatal visits. Thus, a study was undertaken to provide a picture of the psychological well-being and experiences of women at different stages of the perinatal period.

## Methods

A descriptive study design employing both qualitative and quantitative approaches in a successive manner was used. However, this paper is based on qualitative data collected in the first phase of the study. The specific objective was ‘to establish whether lack of social support, uncertainty about survival of self and the baby, anxiety about HIV/AIDS status and testing, and vulnerability/oppression contributed to mental distress during the perinatal period. Data collection for this phase took the form of an interview guide with open-ended questions. The interview guide was used to conduct focus group discussions with participants attending antenatal and postnatal clinics, as well as teachers from local schools. A focus group is defined by [Ritchie and Lewis 27] as a semi-structured group encounter led by a facilitator whose main aim is to elicit information on a specific area of interest. Focus group discussion was chosen as a method of data collection because of its ability to unearth cultural representations and beliefs that mould and affect people’s attitudes and behaviour [27]. The richness of focus group discussion is also grounded in its ability to facilitate self disclosure by participants through the opportunity available to share their perspectives, attitudes, and respond to others’ opinions while also challenging each other’s contributions.

Thirteen questions covered topics such as what it means to be a mother in Zambia, impact of motherhood on spouse, appropriate age to become a mother, and why people experience problems during motherhood, among others. The questions that guided the focus group discussions are shown in Table 1 below.

**Table 1:** Guiding questions

Questions	
1.	What is it like being a mother in Zambia today?
2.	What is a good age to become a mother?
3.	Why do you think people have children?
4.	Does motherhood change women?
5.	What impact does a woman becoming a mother have on her partner/husband?
6.	‘The way a woman goes through pregnancy and birth accounts for her position in the household and in the community’. What is your opinion?
7.	How common is it for people to be unhappy when they are pregnant?
8.	What does motherhood mean to you / what is motherhood about?
9.	What is considered a good mother in our generation?
10.	‘Motherhood is considered to be the happiest moment’. What is your opinion?
11.	Why do you think some people experience problems during motherhood?
12.	‘Life in Zambia has become unaffordable at the moment. Women have to work in order to survive. No wonder some decide to abandon their babies’. What do you think?
13.	‘In this age of HIV/AIDS it is advisable for women not to get pregnant and have children’. What is your opinion?

The data were collected from primary health centres in Zambia and community settings. A total of 159 participants of various ages, and social backgrounds, both married and single, were purposively selected to participate in focus group discussions with 6–12 participants each. Among the 19 focus groups conducted were two groups of older women over the reproductive age, five groups of men, and 12 groups of younger women in antenatal and postnatal periods. Younger women participants were recruited from antenatal and postnatal clinics while male participants were teachers recruited from local schools. Older women were drawn from local church organisations. They were selected to reflect a diversity of cultural views.

The focus group discussions’ aim was not to arrive at consensus, but to capture participants’ diverse views from which to draw common themes. All participants were over 18 years old. Young mothers below the age of 18 were not represented for ethical reasons. Purposive sampling technique was used taking into account the limitation in the availability of participants who satisfied the eligibility attributes. However this was not applicable to male participants and older women. The limitations of purposive sampling technique include inability to make generalisations to any other population except the sample studied, and does not specify the chances that any person or unit on whom the study is based will be included in the sample [27].

On the other hand, the strength lies in the likeliness to access the population that are most likely to poses the attributes the researcher is interested in. It is the most appropriate sampling method in situations where there are few elements in the population which have the eligibility attributes [27]. All participants approached accepted to participate in the discussions and no refusals were recorded.

Analysis of the data took the form of thematic analysis informed by [Braun and Clarke 28]. Data from the tape recorded group sessions were transcribed verbatim. The verbatim were then compared with the data contained in the notes. Transcripts were checked for any mistakes that could have been made during the transcriptions as advised by [Creswell 29]. The responses were coded by colouring responses from the various groups of participants differently. The data was repeatedly compared with the codes to prevent a shift in the meaning of the codes during the process of coding. Long table analysis as a low-technology option [30] was used where responses to the questions from the various groups of participants, in different colour codes were pasted onto the flip charts. Each question was on a different flip chart followed by responses from the different groups of participants identifiable by the colour code. Long table analysis facilitated familiarisation with the data. Theme identification was then done by searching across the entire data set as recommended by [Braun and Clarke 28]. The subsequent stage of description involved showing explicit meanings of the data on semantic level, after which interpretation was undertaken.

Institutional review board approval was obtained from Leeds Metropolitan University, Faculty of Health Research Ethics Sub-committee and the University of Zambia, School of Medicine’s Research Ethics Committee.

Clearance was also sought from the Lusaka District Health Management Team and the Lusaka District Education Office in order to gain access to health and education facilities that participated in the study. Information concerning participation and consequences of the study was availed to all the participants. Participation in the study was voluntary and based on informed consent. Participants were informed that the information collected was anonymous and confidential, but it could be shared with medical personnel in case of risk. Participants were informed of their right to withdraw from participation any time without prejudice. Anonymity was maintained. All names used were pseudonyms.

## Results

Results emerged from analysis of data obtained through focus group discussions involving groups of younger and older women, and groups of men as participants. Their perceptions of motherhood were discussed and conclusions on how these contributed to mental distress in the perinatal period were drawn. The responses represent the three groups of participants (younger women, older women, and male participants) in order to capture the different opinions.

### Socio-demographic characteristics

One hundred and fifty nine participants took part in the focus group discussions. The age of both male and female participants ranged between 18 and 45 years. Female participants were either attending antenatal or postnatal clinics or above the reproductive age, while male participants were teachers. Various themes emerged including how the themes positively and negatively influenced women’s psychological well-being during the perinatal period. This paper focuses on those factors that women thought negatively influenced their psychological wellbeing during the perinatal period as outlined below.

A negative perspective of motherhood was highlighted resulting from various common experiences among women attending antenatal and those attending postnatal clinics in the group discussions. The most pervasive theme was unhappiness common to most of the women participants. The experiences women considered negative are outlined below and include: Uncertainty about survival of self and the baby; anxiety about HIV status and testing; Lack of support; and vulnerability/oppression.

### Uncertainty about survival of self and the baby

Most women (both antenatal and postnatal) felt that motherhood, especially during the antenatal period, was a source of worry because women could not foretell whether they would go through the process of childbirth without complications, and survive it. Male participants as well as older women acknowledged this view. Below are extracts from younger woman and male participants respectively.

"*YOUNGER WOMAN: You are unhappy because you don’t know how and whether you will deliver okay or whether the child will be okay. You can’t get happy until you give birth because you don’t know what will come.*"

"*YOUNGER WOMAN: For first pregnancy it is difficult to be happy because you are anxious about the process of labour whether you will survive or whether the baby will be healthy and safe.*"

"*MALE PARTICIPANT: When they are pregnant, they are closer to the grave –They are scared of death.*"

Worry regarding survival from childbirth is a common feature in Zambian culture. The estimated maternal mortality rate in Zambia is 605 per 100 000 live births as compared with 17 per 100,000 in the United States [31], and thus women cannot guarantee their survival from childbirth.

### Anxiety about HIV/AIDS status and testing

As well as being worried about how they would manage to go through child birth, women experienced anxiety and worry because they were uncertain about their general health status. Women in most groups mentioned that it was mandatory for all pregnant women to be tested for the HIV, and that if they declined to get tested, medical staff would not attend to them during delivery. This appeared to be a source of worry for women as exemplified in the statements below from participants.

"*YOUNGER WOMAN: It is very worrying to test, but we have no choice.*"

"*YOUNGER WOMAN: Others are scared to come to the clinic because they are scared of being found HIV positive.*"

"*OLDER WOMAN: And….if they refuse to take a test, they won’t be delivered at the time of delivery.*"

The above view was especially pervasive in pregnant women who were waiting for testing, but postnatal women spoke of it as having been a source of worry for them as well, probably because they were not guaranteed a negative test result next time they would be due for testing. In Zambia, while HIV testing for pregnant women is based on “voluntary counselling and testing”, women may be refused prenatal treatment if they have not been tested.

### Lack of support

Some of the pregnant women were not supported by their partners. Denial of paternity by the man responsible for the pregnancy was a source of distress. Participants worried about how they would manage to care for their babies in cases where the man refused to acknowledge the pregnancy. This was especially common with women whose pregnancies were unplanned and whose relationships with their partners were not officially recognised.

Some participants highlighted refusal of responsibility by men as one of the sources of worries experienced by women in the perinatal period, while feelings of helplessness were also prevalent in married women although some of the participants believed they were only common in single women. Men, married or not, were said to desert their pregnant wives and partners respectively, for other women. From women’s views, desertion of women by men was prevalent during pregnancy as well as after child birth.

"*YOUNGER WOMAN: If you are not married, it’s worrying especially if the man refuses responsibility of the pregnancy.*"

"*YOUNGER WOMAN: Some men abandon you and start seeing other women because you are pregnant, especially if you have two children. They don’t spend time with you. They go for other women.*"

"*OLDER WOMAN: Men are same, ee..(yes) they run away from their pregnant wives, even when the woman delivers, they will still run away, but we just have to endure.*"

Most women attributed desertion by their partners to the change in women’s focus when they became mothers. They transferred their attention from their partners to their new baby.

This shift in attention made their partners feel less valued and used it to rationalise their engagement in extra marital relationships. Statements below, from participants provide testimony to the dynamics around desertion of women by men especially after childbirth.

"*OLDER WOMAN: Yes, our focus is on the child and the man gets less care….then he starts looking for other women.*"

"*YOUNGER WOMAN: The coming of the baby chases the men away, but what do we do? Children are also important.*"

"However, men revealed opposing views. For them, the arrival of a child harnessed their relationship with their partner and reduced the chances of them divorcing their women."

"*MALE PARTICIPANT: There is happiness and love for the wife after a child. Love towards each other, love is first and children second.*"

"*MALE PARTICIPANT: The bond grows more between the wife and husband, causes of divorce are reduced.*"

"*MALE PARTICIPANT: Children brighten the relationship with your wife.*"

For women who get pregnant while they are still with their parents or guardians, from men who may still be in a similar situation (living with their parents or guardians), the environment was said to be a source of stress.

They were forced by their parents to move in with the men responsible for their pregnancy. Being in a foreign home, they suffered mistreatment and had restrictions in what they were allowed to do.

"*YOUNGER WOMAN: Sometimes, a man’s relatives mistreat you especially bakakufwitila (if they force you on them) – So umankala (you are) unhappy.*"

"*YOUNGER WOMAN: If you are being kept, you will be forced to overwork and can’t take rest when you want.*"

Another facet of the distress for women was a lack of support when the husbands deserted their pregnant wives, as opposed to just having an extramarital relationship. However, it is not uncommon for the man to still be living within the household but not providing financial support to his family. As most women were dependant on their husbands for financial sustenance, lack of material support raised worry for women, about the welfare of their family. Besides men being a source of unhappiness through abandonment of their families, they were labelled as a source of fear for contracting disease due to their promiscuous behaviour. They were said to engage in unprotected sex with other women, from whom they contracted diseases like HIV which in turn they transmitted to their wives.

"*YOUNGER WOMAN: If the man abandons you, you can’t afford to buy food. Especially if one is breastfeeding because you need to eat frequently. Kuchepa kwa ndalama (inadequacy of finances) especially when you are dependent on the man. When he comes back he comes with HIV.*"

"*YOUNGER WOMAN: Motherhood these days is scaring because of the prevalence of diseases like HIV. It is very difficult because we are suppressed by men. Men bring HIV to us because they like sleeping around.*"

"*OLDER WOMAN: This generation is very unfortunate, because when a man deserts his wife, he comes back infected with HIV and he gives it to his wife. We didn’t have HIV in our time, so it was not as bad.*"

### Vulnerability/oppression

Participants interviewed expressed their vulnerability and oppression through various statements. There was widespread agreement among participants that they lack decision-making powers and control over their own lives. The common thread that can be traced through participants’ statements is the dominance of the men. Women felt decisions to do with having children were better left for the man to undertake. This is evident in the statements below.

"*YOUNGER WOMAN: It is not possible to stop bearing children because of HIV/AIDS. It might be easy for single women but for us married women, the man won’t cooperate.*"

"*OLDER WOMAN: In married relationships, men do not compromise for the woman to stop bearing children; women are scared of making their own decisions even during life threatening situations, for example, bleeding during pregnancy leading to death.*"

These statements were shared by other participants as articulated by one of the younger women below.

"*YOUNGER WOMAN: It is true what she has said, what they tell us about HIV/AIDS is true but we live in different homes. But like my colleague has said, as women we might go family planning but it brings problems in the home in future. The man won’t allow you.*"

From the statement above by one of the older women, vulnerability and oppression was also represented by women lacking power to make decisions about their health, even in serious situations like bleeding, which might result in fatality. Women also lack control and power when it comes to decision making about HIV testing, even with the current high prevalence of the HIV/AIDS. Numerous participants commented that men were uncooperative with issues to do with HIV testing, putting women at risk of contracting the virus. This is evident in participants’ statement below.

"*YOUNGER WOMAN: Other men refuse to go for testing. They take medicine without their wives knowing.*"

"*YOUNGER WOMAN: You need to be tested for HIV. But men refuse to be tested, they say if they found you negative, it means I am also negative.*"

"*OLDER WOMAN: They will get tested secretly and when they are positive, they won’t tell anyone.*"

The above statements represent one of the ways in which women in the population under study may be experiencing oppression by men. Vulnerability and oppression was also evident through lack of support that women experienced when they were pregnant and after delivery. Denying women material as well as emotional support as evident above may be regarded as a way of oppressing women in the home. Additionally, many women argued that it was easier for single women to make decisions about reproduction than it was for married women. It was however acknowledged that being single had its challenges.

### Single motherhood as a source of worry

Motherhood was also considered an unhappy experience if a woman was not married. What seemed to pervade these discussions was the inseparability of marriage from motherhood as the two appeared throughout the discussions as if they were synonymous and marriage as a road to happiness. A woman had to be married before she had children as a prerequisite to happiness.

"*MALE PARTICIPANT: Those who are well married will be happy but those impregnated by a boyfriend will be unhappy because of the implications.*"

"*OLDER WOMAN: A woman is happy when there is harmony in the home especially if the husband cares. A woman without a man has problems.*"

The assertions given above were common to all the different group interviews, although they appear to contradict earlier sub-themes about men as a source of negative emotions which have been asserted mostly by women groups, and hence reinforce the notion of the complexity of motherhood as an experience.

"*YOUNGER WOMAN: Being a mother is going through hardships, enduring them. Ukukwata imisula (Being tolerant).*"

"*YOUNGER WOMAN: It is a difficult experience but we have to get ready for it. Mothers are required to be strong. It is a hard job taking care of the children.*"

"*OLDER WOMAN: Motherhood is not easy, marriage (pause) is not also easy, but as women, we are taught to be strong, and we should hold on.*"

A sense of endurance is commonly inculcated in women in Zambia. A strong woman is one who possesses the ability to withstand problems in her marriage, including extramarital affairs by her husband. The latter is a common practice; men’s extramarital affairs are regarded as justifiable while women’s (extramarital affairs) are considered unacceptable and warrant instant divorce.

## Discussion

There is widespread lack of awareness about women’s experiences during the perinatal period and how they influence their psychological wellbeing [3]. It is important for Zambia to generate local evidence regarding the nature and prevalence of maternal mental health problems. Unhappiness was one of the common features ascribed to motherhood by participants in the study. Motherhood was considered to fall short because of the various negative experiences encountered during the perinatal period. Women experienced distress during pregnancy arising from a sense of uncertainty about the impending process of childbirth. They were unsure as to whether they would go through the process of childbirth and survive it without experiencing any complications. This uncertainty may originate from the fact that risks associated with pregnancy and childbirth in developing countries are still considerably high.

Worldwide, about 600, 000 women die annually from complications arising from pregnancy and childbirth and most of these are said to take place in developing countries [26]. The risk of maternal death in some developing countries has been estimated to be as high as one in every seven women (lifetime risk) and as low as one in five thousand in some industrialised countries [32].

Some of the factors attributed to this high rate of maternal mortality include poverty, women’s limited access to education, poor nutrition, lack of access to quality health services, and lack of financial means to pay for the much needed health services [26]. Probably this is more reason why a woman who has just delivered is greeted by a special greeting in one of Zambia’s common languages, ‘mwapusukeni’, meaning ‘you have survived’. In this case, it may be considered rational that these women would worry about their survival while in their transition period to labour considering the high probability of a woman dying or the pregnancy ending in complications during this period.

In addition to anxiety related to the process of childbirth, most women revealed that they experienced stress and anxiety because they worried about their health status, which was mainly in relation to the prevalence of HIV/AIDS in Zambia. The Central Statistical Office of Zambia [33] reports the HIV prevalence rate of 14 percent in the 14 to 49 age group in Zambia. The women interviewed highlighted being subjected to Voluntary Counselling and Testing (VCT) of the HIV so that if found positive, they would then be given medication for Prevention of Mother To Child Transmission (PMTCT) of the virus. However, in reality the counselling was not voluntary because women had to be tested regardless of whether they wanted it or not. If on the other hand they tested negative, they would be provided with information on how to keep themselves safe. The mandatory testing of HIV instilled fear and worry in women thinking their lives would be shortened if they tested positive.

It is pertinent to mention that if these women were being subjected to VCT regardless of whether they were willing or not, voluntarism can be said to be compromised because of the mandatory nature of the VCT. This may exacerbate the distress experienced by women especially in an environment with fragmented mental health interventions for women in the perinatal period.

Most of the studies conducted in relation to people’s reactions with regards their HIV status have ignored the mental health of women during the perinatal period [34], even though it is evident that women who have tested positive for the HIV have been found to exhibit depressive symptoms [35]. However, some improvements have been recorded as the recognition of the impact of HIV/AIDS on the psychological aspect of people in southern Africa is being acknowledged. The emerging mental trauma caused by HIV during the perinatal period affects not only the individual but the family, and the community as whole [36]. This illuminates the need for investigating the psychological well-being of those pregnant women who have just undergone VCT and those who have just been given their results and tested positive. Immediate screening for mental health problems might yield results with a clearer picture of women’s mental well-being at this time and only then can suitable interventions be put in place to reduce distress associated with HIV in women.

A study by [Olley et al., 37] found high prevalence of mental health problems in people who were newly diagnosed with HIV while [Mfusi and Mahabeer 38] found increased incidence of depression in HIV infected women.

Furthermore, women blamed their unhappiness on men who denied responsibility of the pregnancy as well as those who deserted their partners during pregnancy or after the baby was born. According to women in these discussions, it is assumed that the reason why men deserted their partners was the shift in women’s focus of attention and care from their partners to their children, which left men feeling neglected and drove them into seeking affection from other women.

Research studies in support of this assertion highlight a general reduction in marital satisfaction after childbirth as a result of the effects that the arrival of the baby imposes on the behavioural interaction of the couple, including the need for the couple to learn and adopt new problem-solving strategies that may be appropriate to their new situation [39,40]. While [Cowan and Cowan 41] blame the competition between parental and partnership roles for the decline in marital satisfaction during the perinatal period, Clulow [42] attributes the decline to what is termed ‘corrosion of time’ which is especially applicable to those couples that have been together for a considerable period of time. Children in this case, may only act to reinforce the decline in marital satisfaction. It is observable from women’s views that desertion by men was prevalent when a woman was pregnant as well as after the arrival of the baby.

While the reasons for desertion during pregnancy are not mentioned, the limitation in the availability of sex when a woman is highly pregnant might be responsible for driving men into engaging in other intimate relationships. In some cultures including Zambia, it is tolerable for men to take a concubine if they were unhappy in their marriage or if they are not being satisfied sexually [43]. Most of the women interviewed were fully dependent on their partners for financial support.

They were left to struggle economically when their husbands/partners deserted them, which consequently impacted negatively on their psychological well-being. One of the serious threats to maternal well-being during the perinatal period is lack of social support [44].

It should be highlighted at this point that poverty, in this instance due to lack of material support from a spouse, has been identified as one of the contributing factors to psychological distress, with women in low income groups more at risk of exhibiting psychological symptoms related to mood and anxiety than those in higher income groups [45]. Economic dependency by women on their spouses may also put them at high risk of oppression and vulnerability.

It is believed that if a woman is economically dependent on the man, they suffer abuse and oppression silently because they would not be able to provide for their children if they left the man. They leave all decision making about important domestic issues to the man and hardly give their opinion. Over 60 percent of women in Zambia do not take part in decision making in their homes [46]. It is possible that a person who lacks control in what matters to them and lacks an opportunity to make decisions may be considered oppressed, and as such may be more vulnerable to psychological distress arising from oppression than a person who is empowered to make decisions. Further research on the relationship between women’s lack of decision making powers and the levels of vulnerability to psychological distress may provide important insights into what type of strategies would be required to empower women so as to reduce psychological distress during the perinatal period.

Motherhood was also considered an unhappy experience if a woman was single because motherhood and marriage were regarded as inseparable by participants in the present study. This view might have its roots in society’s portrayal of single motherhood as a digression from normality and attaching stigma to it.

Avison [47] agrees that single mothers experienced higher levels of psychological stress than mothers with partners and explains that their vulnerability to psychological distress is because they are in a disadvantaged situation in terms of economic sustenance, being sole providers for their children. Avison [47] dismisses the association of the distress experienced by single mothers to their personal vulnerabilities in the sense that they singly carry almost all the responsibilities of taking care of their children while at the same time, might be experiencing difficulties economically.

However, while acknowledging the implications of single motherhood on women’s psychological well-being, similarities exist in problems experienced by other mothers [48] and evidence from the present study shows that the issues that were responsible for distress experienced by married women during this period of time were similar to those at play in mothers without partners, but future studies which aim to establish the differences in the experiences of psychological distress between the two groups of women is needed in order to provide guidance in the distribution of resources aimed at minimising mental distress in the perinatal period.

WHO [49], reveals that a mother with mental health problems may experience significant self care deficits including inability to maintain her nutrition and hygiene needs, which may increase her vulnerability to infections and anaemia, and may hamper her ability to care for her children. Other studies have revealed that a mother’s mental health is a determinant of the mental health of her children [49-51] in the sense that parental mental distress imposes on children the risk of experiencing general ill health. More over, a higher rate of behavioural, developmental, and emotional problems in children with a mentally distressed mother compared to children in the general community has also been reported. This is also valid for older children [50].

The attachment bond between mother and child is often jeopardised by the presence of mental distress in the mother. As well as the ill parent and her children, the spouse is also affected because care responsibilities frequently fall on the woman’s spouse, which may also result in distress [50]. Mental distress in one parent may subsequently be associated with mental distress and other Psychopathology in the other parent [51]. Therefore, the need to screen women for mental health problems in the perinatal period cannot be overemphasised. Those women identified as experiencing mental health problems should be referred to trained mental health professionals for appropriate interventions.

## Conclusion

The study demonstrates the need to recognise that women experienced distress during the perinatal period arising from a range of problems. Although pregnancy is a normal situation, the particular vulnerability of women should also be recognized during this period. Therefore, improving identification and management of psychological distress should form the core part of the health care provision. Mothers who experience mental health problems during the perinatal period may have difficulties providing appropriate care to themselves and their children.

This may compromise the survival of their children because poor maternal mental health in the mother has been linked to poor growth and higher risk of diseases in children [52]. In addition, maternal mental health is undoubtedly connected to the physical and psychological growth of the child. The importance of a holistic screening programme would subsequently facilitate the provision of a comprehensive package of interventions that would promote women’s psychological well-being during the perinatal period which may even serve to the negative experiences of motherhood earlier described. Therefore intervening in the mental health of the mother will have positive resultant effects for all concerned.

## Competing interests

The authors declare that they have no competing interests.

## Authors’ contributions

LM and TN drafted the manuscript. RD participated in revising the manuscript critically for important intellectual content. All authors read and approved the final manuscript.

## References

[B1] Niven CA Walker A Conception, pregnancy and birth 1996 Butterworth Heineman, Oxford

[B2] Department of Health Women’s Mental Health: Into the Main Stream, Strategic Development of Mental Health Care for Women 2002 DOH, London

[B3] Fisher JRW De Mello MC Izutsu T Tran T The Ha Noi Expert Statement: recognition of maternal mental health in resource-constrained settings is essential for achieving Millennium Development Goals Int J Ment Heal Syst 5 2 (online). Available at: http://www.ijmhs.com/content/5/1/21 Accessed: 24th October, 2011 10.1186/1752-4458-5-2 PMC3226322 21214891

[B4] Currid TJ Psychological Issues Surrounding Paternal Perinatal Mental Health Nurs Times 2005 101 5 40 42 15732493

[B5] Crawford M Unger R Women and Gender: A Feminist Psychology 1986 London, McGraw Hill

[B6] Dulude D Belanger C Wright J Sabourn S High risk pregnancies, psychological distresses, and dyadic adjustment Journal of Reproductive and Infant Psychology 2002 20 2 101 123

[B7] Jomeen J Martin CR Confirming of an occluded component within the Edinburgh Postnatal Depression Scale (EPDS) during pregnancy Journal of Reproductive and Infant Psychology 2005 23 2 143 154

[B8] Watson JP Elliot SA Rugg AJ Brough DI Psychiatric disorder in pregnancy and the first postnatal year Br J Psychiatry 1984 144 453 462 6733369 10.1192/bjp.144.5.453

[B9] O’Hara MW Neunaber DJ Zekoski EM Prospective study of postpartum depression: prevalence, cause and predictive factors J Abnorm Psychol 1984 93 158 171 6725749 10.1037//0021-843x.93.2.158

[B10] Choi P Henshaw C Baker S Tree J Supermum, superwife, supereverything: performing femininity in the transition to motherhood Journal of Reproductive and Infant Psychology 2005 23 2 167 180

[B11] World Health Organisation Community Based Rehabilitation and the Health Care Referral services. A guide for programme managers 1994 World Health Organisation, Geneva Switzerland

[B12] Green JM Postnatal depression or perinatal disphoria? Findings from a longitudinal community-based study using the Edinburgh Postnatal Depression Scale Journal of Reproductive and Infant Psychology 1998 16 143 155

[B13] Webster ML Thompson J Mitchell EA Werry JS Postnatal depression in community cohort.Australian and New Zealand Journal of Psychiatry 1994 28 42 49 10.3109/00048679409075844 8067968

[B14] Jomeen J Martin CR Replicability and stability of the multidimensional model of the Edinburgh Postnatal Depression Scale in late pregnancy. Journal of Psychiatric and Ment Heal Nurs 2007 14 319 324 10.1111/j.1365-2850.2007.01084.x 17430456

[B15] Ingram JC Greenwood RJ Woolridge MW Hormonal predictors of postnatal depression at six months in breast feeding women Journal of Reproductive and Infant Psychology 2003 21 1 61 68

[B16] O’Hara M Swain A Rates and risks of postpartum depression – a meta-analysis Int Rev Psychiatry 1996 8 37 54

[B17] Honikman S Field S Perinatal Mental Health Project: Caring for mothers, caring for the future 2008 University of Cape Town, Western Cape

[B18] Austin MP Perinatal mental health: Opportunities and challenges for psychiatry Australian Psychiatry 2003 11 4 399 403

[B19] Grantham-McGregor S Cheung Y Cueto S Glewwe P Richter L Strupp B Developmental potential in the first 5 years for children in developing countries Lancet 2007 369 9555 60 70 17208643 10.1016/S0140-6736(07)60032-4 PMC2270351

[B20] Cooper PJ Tomlinson M Swartz L Woolgar M Molento C Postpartum depression and the mother-infant relationship in a South African peri-urban settlement Br J Psychiatry 1999 175 554 558 10789353 10.1192/bjp.175.6.554

[B21] Pearce TO Turshen M Death and Maternity in Nigeria African Women’s Health 2000 Africa World Press, Trenton 46 61

[B22] Cox JL Perinatal mood disorders in a changing culture. A transcultural European and African perspective Int Rev Psychiatry 1999 11 103 110

[B23] Affonso DD Mayberry LJ Common stressors reported by a group of child bearing American women Health Care Women Int 1990 11 3 331 345 2391289 10.1080/07399339009515902

[B24] Abiodun OA A validity study of the Hospital Anxiety and Depression Scale in general hospital units and a community sample in Nigeria Br J Psychiatry 1994 165 669 672 7866683 10.1192/bjp.165.5.669

[B25] Guse T Wissing M Hartman W The effect of a prenatal hypnotherapeutic programme on postnatal maternal psychological well-being Journal of Reproductive and Infant Psychology 2006 24 2 163 177

[B26] Maimbolwa MC Maternity Care in Zambia: With Special Reference to Social Support 2004 Stockholm, ReproPrint AB

[B27] Ritchie J Lewis J Qualitative Research Practice. A Guide for Social Science Students and Researchers 2003 Sage Publications, London

[B28] Braun V Clarke V Using thematic analysis in psychology Qual Res Psychol 2006 3 77 101

[B29] Creswell JW Research Design: Qualitative, Quantitative, and Mixed Methods Approaches 2009 3 Los Angeles, Sage Publications

[B30] Krueger AR Casey MA Focus Groups. A practical guide for applied research 2000 3 Sage Publications, Thousand Oaks

[B31] Hogan MC Foreman KJ Naghavi M Ahn SY Wang M Makela SM Lopez AD Lazano R Murray CJL Maternal mortality for 184 countries, 1980–2008: a systematic analysis of progress towards Millennium Development Goal 5 Lancet 2010 (online) Available at: http://www.thelancet.com/doi:10.1016/S0140-6736(10)605180 Accessed: 12th September, 2010 10.1016/S0140-6736(10)60518-1 20382417

[B32] UNICEF Economic and social statistics on the countries and territories of the world, with particular reference to children’s wellbeing 2011 UNICEF, Geneva

[B33] Central Statistical Office Zambia Demographic and Health Survey 2007 CSO, Lusaka

[B34] Collin SM Chisenga MM Kasonka L Haworth A Young C Filteau S Murray SF Factors associated with postpartum physical and mental morbidity among women with known HIV status in Lusaka, Zambia AIDS Care 2006 18 7 812 820 16971293 10.1080/09540120500465061

[B35] Silver EJ Bauman LJ Camacho S Hudis J Factors associated with psychological distress in urban mothers with end-stage HIV/AIDS AIDS & Behaviour 2003 7 421 431 10.1023/b:aibe.0000004734.21864.25 14707539

[B36] Wright J Lubben F Mkandawire MB Young Malawians on the interaction between mental health and HIV/AIDS African Journal of AIDS Research 2007 6 3 297 304 10.2989/16085900709490425 25866175

[B37] Olley BO Gxamza F Seedat S Theron H Taljaard J Reid E Reuter H Psychopathology and coping in recently diagnosed HIV/AIDS patients – the role of gender South African Medical Journal 2003 93 12 928 931 14750493

[B38] Mfusi SK Mahabeer M Psychosocial adjustment of pregnant women infected with HIV in South Africa J Psychol Afr 2000 10 2 122 145

[B39] Cox MJ Paley B Burchinal M Payne CC Marital perceptions and interactions across the transition to parenthood Journal of Marriage and the Family 1999 61 611 625

[B40] Shapiro AF Gottman JM Carrere S The baby and the marriage: Identifying factors against decline in marital satisfaction after the first baby arrives J Fam Psychol 2000 14 1 59 70 10740682 10.1037//0893-3200.14.1.59

[B41] Cowan CP Cowan PA When Partners Become Parents. The Big Life Change for Couples 1992 Basic Books, New York

[B42] Clulow C Partners becoming parents: a question of difference Infant Mental Health Journal 1991 12 3 256 266

[B43] Kaye D Childlessness transformed: stories of alternative parenting. across cultural historical overview of the roles of childless people 2010 (online). Available at: mhtml:file://E\INFERTILITY-DEBRA.mht Accessed: 26th January, 2010

[B44] Elsenbrunch S Benson S Rucke M Rose M Dundenhausen J Pincus-Knackstedt MK Klapp BF Arck PC Social Support During Pregnancy: Effects on Maternal Depressive Symptoms, Smoking and Pregnancy Outcome European Society of Human Reproduction and Embryology 2006 Oxford University press, Oxford 10.1093/humrep/del432 17110400

[B45] Roades LA Biaggio M, Hersen M Mental health issues for women Issues in the Psychology of Women 2000 Kluwer Academic/Plenum Publishers, New York

[B46] Central Statistical Office Zambia Demographic and Health Survey 2001–2002 CSO, Lusaka

[B47] Avison WR Single motherhood and mental health: Implications for primary prevention Can Med Assoc J 1997 156 5 5 18 PMC1232830 9068572

[B48] McLanahan SS Sandefur G Growing up with a Single Parent: What Hurts, What Helps 1994 Harvard University Press, Cambridge

[B49] WHO Maternal Mental Health and Child Health and development http://www.who.int/mental_health/prevention/suicide/MaternalMH/e. Accessed: 06.07.2012

[B50] Najman JM Williams GM Nikles J Spence S Bor W O’Callaghan Le Brocque R Andersen MJ Mother’s Mental Illness and Child Behaviour Problems: Cause-Effect Association or Observation Bias? Journal of American Academia and Child Adolescent Psychiatry 2000 39 5 592 602 10.1097/00004583-200005000-00013 10802977

[B51] Reupert A Maybery D Families affected by Parental Mental Illness: A multiperspective Account of Issues and Interventions Am J Orthopsychiatry 2007 77 3 362 369 17696664 10.1037/0002-9432.77.3.362

[B52] WHO World Health Statistics 2009 WHO, Geneva

